# Associations of Racial Discrimination with Resting-state Network Topology: A Mechanism for Post-traumatic Sensory Disruptions

**DOI:** 10.21203/rs.3.rs-8855028/v1

**Published:** 2026-03-26

**Authors:** Aziz Elbasheir, Leland Fleming, Nathaniel Harnett, Alfonsina Guelfo, Travis Fulton, Timothy McDermott, Timothy Ely, Jennifer Stevens, Negar Fani

**Affiliations:** Emory University School of Medicine; Mclean Hospital; McLean Hospital; Emory University School of Medicine; Emory University; Florida State University; Emory University; Emory University; Emory University School of Medicine

## Abstract

**Background:**

Racial discrimination (RD) is a chronic stressor associated with increased risk for post-traumatic stress disorder (PTSD), a disorder associated with disruptions in neural network organization. However, the neural mechanisms linking RD to PTSD remain unclear. We examined whether RD is associated with network organization metrics, including modularity and clustering coefficient (CC), and whether network metrics influenced associations between RD and PTSD symptoms.

**Methods:**

Ninety adult (age range, 18–62) Black American women recruited for the Grady Trauma Project completed resting-state MRI along with measures of RD, trauma exposure and PTSD symptom severity. Network topology was examined for each of seven resting-state networks; adjacency matrices of each network were used to derive network modularity and CC. Partial correlations were conducted with RD and network metrics with covariates of age, trauma exposure and systemic inequities. Metrics that showed significant associations with RD were entered into moderation analyses with PTSD symptom clusters.

**Results:**

Greater RD exposure was associated with lower CC of the somatomotor network (SMN, r=−.318, *p*= .003). Moderation analysis revealed that RD associated with PTSD re-experiencing symptom severity at relatively lower (≤.48) [B=.58, CI (.22, .95), *t*= 3.20, *p*=.002] SMN CC values; this relationship was not observed at higher CC values (*p*s>.05).

**Conclusion:**

Greater RD linked to lower clustering within the SMN, reflecting a shift toward more distributed network organization. Lower SMN clustering moderated associations between RD and PTSD re-experiencing symptoms. Findings suggest that more frequent RD may disturb the organization of networks responsible for the integration of external sensory and internal visceral signals, which, in turn, may influence the development of PTSD reliving phenomena.

## Introduction

Black individuals are disproportionately exposed to social stressors, including racial discrimination (RD), a known risk factor for the development of post-traumatic stress disorder (PTSD). [1–8]. PTSD is a debilitating psychiatric condition characterized by symptoms of persistent hyperarousal, avoidance, cognitive and emotional alterations alongside intrusive re-experiencing of the trauma, such as memories, flashbacks and nightmares [9–11]. RD is a chronic traumatic stressor for Black Americans, with some reports indicating that ~70% of Black Americans experience RD daily [12–14]. Greater exposure to RD has been associated with more severe symptoms of PTSD [1–8] even after accounting for exposure to non-race-related traumatic stressors. Further, experiences of RD predict PTSD onset and severity in the aftermath of other trauma exposure [6–8]. The mechanisms underlying these outcomes vary, with evidence linking RD to heightened cognitive and physiological effort, stemming from increased threat detection and self-regulatory efforts to alleviate distress[15–17]. Although these strategies may support short-term stress relief, their chronic engagement significantly contributes to psychological strain and, over time, increased risk for poorer mental health outcomes including PTSD [18–20].

The neurobiological pathways through which RD may influence the development of post-traumatic symptoms remain poorly understood. Emerging work suggests that greater RD exposure is associated with alterations in resting state neural networks involved in executive control, salience detection, autobiographical and somatosensory processes [21–25]. RD exposure has been associated with greater resting state functional connectivity (rsFC) within the salience network (SN), which engages in the detection of important environmental cues [21–24]. RD has been associated with stronger coupling between the amygdala, a central brain region for threat detection, and the thalamus [21, 22] as well as broader alterations in SN connections with sensory systems; seed-to-voxel analyses with core SN nodes (e.g., insula, amygdala) indicate that RD is associated with reduced SN rsFC with the somatomotor network (SMN; involved with integration of bodily sensations with motor processes) [23, 24] and increased SN connectivity with the visual network [23] and posterior aspects of the default mode network (DMN), which engages during mind-wandering and autobiographical processes [22]. To date, one study has examined discrimination-related whole network alterations across different racial and ethnic groups [25]. Overall, participants who reported more frequent discrimination exposure (inclusive of, but not limited to race) displayed greater DMN to SMN connectivity, as well as lower rsFC between the central executive network (CEN) and the SMN and visual networks [25]. In Black participants, discrimination associated with greater within-network connectivity of the DMN and CEN [25]. Collectively, findings could suggest greater network resource expenditure in relation to various types of high-effort coping (e.g., vigilance for racial threats, rumination, emotion suppression, processes modulated by SN, DMN and CEN). However, no related clinical or behavioral data were available to confirm this.

Neural network studies of PTSD similarly show alterations across large-scale rs-networks involved in executive control, autobiographical processing and salience detection [26–38]. As compared to trauma-exposed controls, those with PTSD show greater SN within network connectivity [36, 37] as well as SN-DMN connectivity [36, 38] in which these changes in SN connectivity are related to worse overall symptom severity [36]. Further, diminished within-network CEN connectivity has also been associated with greater overall PTSD symptom severity [26, 27]. Mixed findings have been observed within the DMN, with some studies showing lesser DMN within-network connectivity in association with more severe re-experiencing [31], avoidance [32] and dissociative symptoms [33] whereas other studies show greater DMN within-network connectivity in relation to re-experiencing [34] and overall PTSD symptom severity [35]. Moreover, greater between-network connectivity of the DMN has been associated with worse PTSD symptoms. Greater DMN to CEN network connectivity has been associated with greater dissociation [28, 29] and re-experiencing symptom severity [30] whereas greater DMN to SMN network connectivity has been associated with worse re-experiencing symptoms [39, 40]. Together, these findings suggest that PTSD is characterized by CEN, DMN and SN network disruptions that have been most consistently linked to re-experiencing and dissociative symptoms.

An increasing number of studies have examined network organization changes in relation to PTSD phenomenology [31, 41–46]. Identifying the intrinsic organizational patterns of rs-networks illuminates, at baseline, the degree of coordinated communication within networks, balance of communication between networks and how these patterns may relate to symptoms. For example, two networks may show similar connectivity patterns but differ greatly in the efficiency of communication or how well they maintain functional boundaries both within and between networks [47, 48]. As such, examining alterations in topological architecture of rs-networks may elucidate how trauma reshapes functional network organization. Network cohesion metrics, such as modularity [49, 50] and clustering coefficient (CC) [51], offer information about the intrinsic balance of specialization and communication within and across networks. This is critical for understanding network behavior and function [47, 48]. Modularity quantifies the degree to which a network is organized into distinct, densely connected communities or “modules.” Highly modular networks are those with dense within-module connections and sparse connections to other modules [49, 50]. Clustering coefficient measures how interconnected a given node’s neighbors are to each other [51]. High clustering indicates densely interconnected neighboring nodes within the network; indicating more efficient local communication and greater resistance to failure from any one node’s damage or removal. While elevated clustering can give rise to the emergence of modular structure, these metrics capture distinct organizational principles that are critical for understanding the behavior of rs-networks.

PTSD phenomenology has been linked to less specialized and efficient rs-network organization including the DMN, SN, SMN and visual networks [31, 41–46]. Compared to trauma-exposed controls, veterans with PTSD showed lower SN CC values [41]. Among veterans with PTSD, worse overall PTSD symptom severity [42] and worse re-experiencing symptom severity [31] has been associated with less efficient within-network connectivity of the DMN. Compared to controls (both trauma and non-trauma exposed), some studies observed that PTSD groups show alterations in functional organization of sensory networks. Specifically, greater modularity of the visual network [43] and greater system segregation of the SMN [44, 45] have been observed in PTSD populations compared to controls. Reduced clustering coefficient of the SMN has been associated with elevated re-experiencing [46]. Overall, findings suggest that PTSD is characterized by less efficient communication of networks associated with autobiographical and sensory processes as well as threat detection which may, in turn, exacerbate both PTSD symptom severity, particularly re-experiencing.

Despite growing evidence linking trauma exposure to alterations in network topology, much less is known about how RD affects rs-network organization. RD, as a chronic stressor, may alter intrinsic rs-network organization, promote prolonged engagement of threat-related networks and compromise regulatory control at rest. These baseline network alterations may then confer risk for PTSD by increasing hyperarousal, threat related intrusions and impairing emotion regulation more generally. As such, we explored associations of RD with resting-state network topology in a population of trauma-exposed Black women who participated in a long-standing PTSD study, the Grady Trauma Project. We used graph theory tools to first examine the relationship between RD exposure and network topology, examining network modularity and CC, metrics of network cohesion that have been linked to PTSD [39–44, 46, 52, 53]. Next, since rs-network cohesion varies across individuals, differences in intrinsic network organization (e.g., high vs low modularity and/or CC) may condition the strength of the association between RD and PTSD phenomenology. Accordingly, we investigated network modularity and/or CC as moderators to determine whether the relationship between RD and PTSD differs as a function of network cohesion.

## Methods

### Participants.

Ninety women who self-identified as Black aged 18 – 62 years [mean (SD) age=38.49 (11.26) years] were recruited as part of Grady Trauma Project (GTP), a collection of studies investigating biomarkers and interventions for trauma-related disorders (including MH101380, HD071982, MH071537, and MH094757) [54]. Due to some GTP studies recruiting only or primarily women (e.g., MH101380, HD071982), and the higher rate of women recruited in GTP studies overall, we restricted analyses to women only. Our sample was recruited in general medical clinics (obstetrics/gynecology, diabetes, and primary care) of a publicly funded hospital located in Atlanta, Georgia. Participants were screened for prior trauma exposure (detailed in Clinical Assessments), which was the primary inclusion criterion. Exclusion criteria were the following: physical or medical conditions that would prevent MRI scanning (e.g., metal implants); current or past diagnosis of schizophrenia or other psychotic disorders; medical conditions that may contribute to cognitive impairment (i.e., dementia); any history of head injury or loss of consciousness for longer than five minutes; history of neurological disorder. All participants provided informed consent before enrollment into studies. Clinical assessments of trauma exposure, RD, and PTSD symptoms were administered. Eligible participants participated in a magnetic resonance imaging (MRI) scan on a separate visit. Clinical and demographic characteristics of these participants are described in Table 1. Mean imputation was used to address missing data points; we calculated the mean of monthly income and education level for those participants and replaced missing values with each participant’s mean monthly income and education level. Oversight of this study was provided by the Emory University Institutional Review Board and Grady Research Oversight Committee.

### Assessments.

Participants completed the Experiences of Discrimination (EOD) questionnaire, a widely used measure of RD with good psychometric properties [55]. Participants are asked to identify lifetime experiences of unfair treatment due to their race, skin color, or ethnicity across different settings (e.g., medical, retail, law enforcement); a summed score representing the number of types of RD experienced was used in analyses (score range = 0–9) [55]. The Traumatic Events Inventory was used to quantify the number of different types of traumas participants experienced during the lifetime [56]. The PTSD Symptom Scale [PSS; score range = 0–51] was administered to examine the presence and severity of PTSD symptoms within the last two weeks [57]. The Childhood Trauma Questionnaire (CTQ) was used to quantify exposure to childhood maltreatment[58]. Systemic inequities were assessed via a composite variable created that included information on financial instability (i.e., monthly income), and housing instability (e.g., evicted from house or apartment), further detailed in the **Supplement**.

### MRI Acquisition and Image Processing.

Magnetic resonance imaging was conducted on either of 2 identical 3T scanners (MAGNETOM TIM-Trio; Siemens) at Emory University, with identical acquisition parameters. Functional MRI data preprocessing and quality assessment were performed with the CONN toolbox, version 21.a (CONN) [59] using their default pipeline. Preprocessing included functional scan realignment, slice timing correction, co-registration to MPRAGE (magnetization-prepared rapid acquisition gradient echo), spatial normalization, and smoothing with a full-width half-maximum isotropic gaussian kernel filter of 8 mm. Functional scans were subjected to motion outlier identification using the Artifact Detection Toolbox (https://www.nitrc.org/projects/artifact_detect/). Functional and structural images were normalized to Montreal Neurological Institute space (MNI152). Principal components filtering was used to identify anatomical noise (10 components for white matter, 5 components for cerebrospinal fluid); anatomical noise was included as a second-level covariate in statistical models.

### Network Construction, Modularity and Clustering Coefficient Analysis.

The Brainnetome Atlas [60], a high-resolution, connectivity-based parcellation was used to parcellate the brain into 210 cortical regions of interest (ROIs) within seven networks defined by the Yeo cortical atlas, which included the DMN, SMN, CEN, ventral attention network, dorsal attention network, limbic network and visual network [61]. Time series from each ROI were then obtained by averaging the time series of each of its voxels. Pearson correlation coefficients were calculated for all pairs of ROIs generating a 210 × 210 connectivity matrix. A Fisher’s-Z transformation was then applied to the correlation matrices to improve normality [62]. To focus on the most robust functional connections and minimize influence of weak or potentially spurious connections, the connectivity matrix was binarized using a density threshold of 20%, such that only the strongest 20% of connections were retained and all weaker connections were removed. These binarized matrices were used to create unweighted, undirected whole-brain graphs for each participant, from which modularity and clustering network metrics were derived using the Brain Connectivity Toolbox (BCT; http://www.brain-connectivity-toolbox.net/) and used in primary analyses[63]. Modularity describes differences between the number of connections within modules from the number of connections across modules [49]. Modularity was defined as $\sum_{i=1}^{m}\left(e_{ii}-a_{i}^{2}\right)$ where $e_{ii}$ is the fraction of connections that connect two nodes within module $i,\;a_{i}$ is the fraction of connections connecting a node in module i to any other node, and m is the total number of modules in the network [49]. We used a spectral algorithm [50] to identify the partition that maximizes modularity for each participant at our defined threshold. CC, a metric that describes the fraction of triangles around an individual node [51], was defined by the following equation: $C_{i}=\frac{2e_{i}}{k_{i}\left(k_{i}-1\right)}$, where $e_{i}$ represents the number of connections the neighbors of node i make with each other and $k_{i}$ represents the degree of node i where $k_{i}\left(k_{i}-1\right)$ represents the maximum number of possible edges, or connections between is neighbors.

### Statistical Analysis.

Modularity and CC values were extracted for each of seven networks (visual, SMN, dorsal attention network, ventral attention network, limbic network, frontoparietal network and DMN) to quantify intrinsic network organization. Using SPSS version 27 (IBM Corp.) partial correlation analyses were conducted to examine associations between lifetime exposure to RD and modularity and CC of resting-state networks controlling for age, adult trauma exposure, childhood trauma exposure and systemic inequities at a Bonferroni-corrected *p* = .007 (*p*=.05/7 networks) for each of the two analyses. For networks showing significant associations with RD, network cohesion metrics were entered into three moderation models to assess whether network topology moderates the relationship between RD and PTSD symptom clusters (re-experiencing, avoidance/emotional numbing, hyperarousal) while controlling for age, adult trauma exposure, childhood trauma exposure, systemic inequities and scanner type; statistical significance was defined at Bonferroni-corrected threshold of *p* =.017 (*p*=.05/3 symptom clusters).

## Results

### Associations of RD with trauma exposure, PTSD, systemic inequities, and age.

Frequency of RD (EOD total score) ranged from 0 to 8 (mean = 2.39, SD =2.21). As expected, EOD total significantly correlated with PTSD symptoms (r = .224; *p* = .033), childhood maltreatment (r= .221, *p* = .037), lifetime trauma exposure (r = .364 *p* < .001), and age (r= .381, *p* < .001). RD was not significantly associated with the systemic inequities’ composite variable (r= .104, *p* = .329).

### Associations of RD with Network Topology.

**Figure 1:**
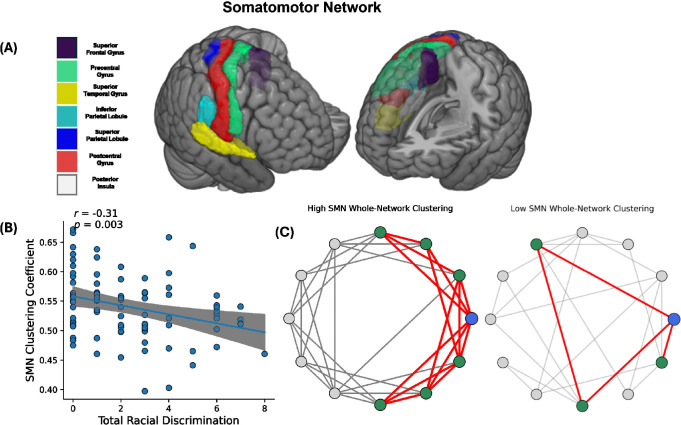
**(A)** Somatomotor network regions, as defined by the Yeo atlas[61]. **(B)** More exposure to racial discrimination associated with less clustering of the somatomotor network after controlling for age, childhood and adult trauma exposure, systemic inequities and scanner type (r = −.318, *p*=.003). **(C)** A representative schematic highlighting differences between a highly clustered SMN network versus a network with low clustering. Edges connecting node *i* to its neighbors and edges between neighbors (forming triangles) are shown in red to illustrate local neighborhood clustering. **Left:** SMN network characterized by dense local interconnections among neighboring nodes. **Right:** A low-clustered SMN network with an equivalent number of nodes and edges but reduced local interconnectedness. Node *i* (blue) and its immediate neighbors (green) are highlighted to illustrate differences in local clustering.

Participant-level heat maps of modularity and CC across the seven functional networks demonstrated inter-individual variability. Participants were ordered by SMN values to visualize cross-network patterns **(see Supplemental Figure 1).** At our statistical threshold, RD exposure (EOD total) was not significantly associated with modularity of any of the seven networks (see Table 2A). However, greater RD exposure correlated with lesser CC of the SMN (see Table 2B, Figure 1A and 1B; r = −.318 *p* = .003).

### Moderation Analyses with PTSD Symptoms.

**Figure 2:**
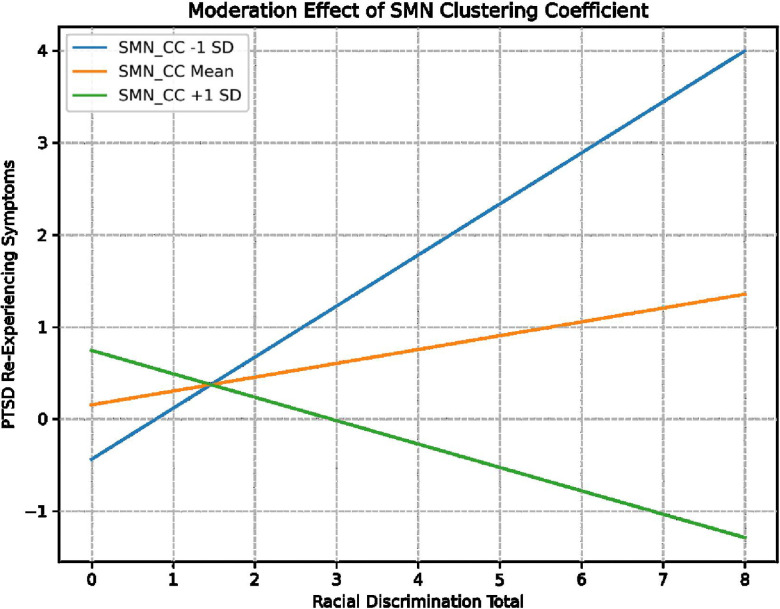
Somatomotor Network (SMN) clustering coefficient (CC) moderates the association between RD and re-experiencing, such that the effect of RD on re-experiencing was stronger at lower (≤.48) [B = .58, CI (.22, .95), *t* = 3.20, *p* = .002] clustering coefficient values but not mid-range (.54) [B = .30, CI (−.02, .62), *t* = 1.85, *p* = .07] or high SMN clustering coefficient (≥.60) [B = −.003, CI (−.51, .50), *t* = −.01, *p* = .98].

Three moderation analyses were conducted with RD (EOD total) as the predictor, PTSD total and PTSD symptom clusters (hyperarousal, avoidance/numbing and re-experiencing) as the outcome variables and SMN CC as moderator, given results from network analyses. Overall models of avoidance F (7,73) = 4.64, *p* = <.001, R^2^ =.58, re-experiencing F (7,73) = 3.00, *p* = .005, R^2^ =.49 and hyperarousal F (7,73) = 2.85, *p* = .008, R^2^ =.48 were significant; model statistics provided in **Supplemental Table 1.** For re-experiencing symptoms, there was a significant main effect of RD [β = 3.2, Cl (.38,6.12), t = 2.25, *p* = .02] as well as a significant interaction of SMN CC and RD [β = −5.4, Cl (−10.9., −.004), t = −1.9, *p* = .014] after controlling for age, adult and childhood trauma exposure, systemic inequities and scanner type (see Table 3A). Analysis of simple slopes indicated that SMN CC positively moderated the association between RD and re-experiencing, such that the effect of RD on re-experiencing was stronger at lower SMN CC (≤.48) [B = .58, CI (.22, .95), *t* = 3.21, *p* = .002], but not mid-range (.54) [B = .30, CI (−.02, .62), *t* = 1.85, *p* = .07] or high CC (≥.60) [B = −.003, CI (−.51, .50), *t* = −.01, *p* = .98] of this network (see Figure 2; Table 3B). No significant moderation was observed with other PTSD symptom clusters, as detailed in Supplement.

## Discussion

We used a graph theory approach to examine potential associations between RD exposure and topology of rs-networks in a sample of trauma-exposed Black women. We also tested whether network alterations moderated the relationship between RD and PTSD symptom severity. We found that greater RD exposure linked with diminished clustering coefficient of the SMN, and this, in turn, moderated the relationship between RD and PTSD re-experiencing symptom severity. Altered connectivity has been previously observed within large-scale resting-state networks in relation to RD and PTSD [21–38, 64]; greater RD exposure was associated with altered rsFC among key sensory-affective circuits including amygdala-thalamus [21, 22], insula-somatosensory cortex [24] and visual pathways [23]. Our group also observed that greater exposure to RD associated with disrupted interoceptive network connectivity in the face of threat-related cues, and these disruptions were positively associated with derealization symptom severity [65]. However, these studies largely focused on functional connectivity between specific regions rather than intrinsic functional organization of whole rs-networks and how these networks function as integrated, coordinated systems. The present findings extend earlier research, indicating RD may disrupt the intrinsic network structure of sensory networks, providing a putative network-level mechanism linking RD and altered sensory rsFC. Reduced functional specialization within the SMN may undermine efficient sensorimotor processes including less stable coordination among primary motor and somatosensory regions. This, in turn, may impair processing of sensory signals of relevance for emotion regulation, thereby increasing vulnerability for the development of PTSD re-experiencing symptoms in the aftermath of trauma.

Our findings suggest that RD may be “embodied” via alterations in the structure of the somatomotor network. Sensory and motor features of non-trauma memories are thought to be integrated within the DMN (particularly anterior aspects) where they become abstracted and temporally contextualized [66, 67]. This integration allows for memories to be recalled as past events without involuntary reactivation of raw sensory and bodily states. In contrast, traumatic memories are fragmented and de-contextualized [66–68]. Some neurobiological trauma frameworks indicate greater brainstem/midbrain engagement during trauma encoding, as well as hyperconnectivity between the SMN with posterior aspects of the DMN [66–68]. As a result, traumatic memories may re-emerge as raw sensory and motor memory fragments, giving rise to intrusive sensory flashbacks and other high-arousal re-experiencing of trauma memories. A growing body of trauma research has identified sensory processing alterations as a core feature of PTSD [69]. A recent review demonstrated that PTSD is associated with widespread disruptions in sensory networks as well as altered communication between sensory, autobiographical and executive control networks, suggesting alterations in both local and large-scale network organization. The present study extends earlier findings, linking RD to disrupted functional cohesion of the SMN. Lower SMN clustering may represent a neural signature of embodied racial stress, which in turn, may predispose individuals to sensory re-living of trauma.

Somatosensory disruptions are a common response to RD; increased muscle tension [70, 71] and chronic pain are linked to this stressor [72–74]. As such, RD-related alterations in the SMN may also intersect with aberrant nociceptive processing commonly observed following chronic trauma exposure [75–77]. Although not directly tested here, reduced local specialization within the SMN may contribute to disruptions in bodily regulation, including elevated muscle tension and pain processing, providing a potential neural mechanism linking RD to chronic pain and somatic symptoms in Black populations. Beyond somatic dysfunction, SMN clustering has also been proposed as a marker of age-related health outcomes [78–81]. Functional segregation of the SMN follows an inverted U-shaped trajectory across the lifespan, with age-related declines associated with poorer motor coordination and health, suggesting network segregation as a potential indicator of accelerated brain aging [79, 80]. Within this context, the present findings may offer preliminary evidence linking RD to alterations in functional network organization consistent with accelerated brain aging, aligning with prior work demonstrating strong associations between RD and biological aging [82–85] in which alterations in rsFC act as mediating mechanism [54]. As such, the SMN also represents a putative mechanism linking racialized traumas like RD to somatic health and age-related vulnerabilities.

These findings lend credence to the value of somatically-oriented therapeutic strategies in racially minoritized populations, including therapies that focus on breath, movement and bodily awareness [86, 87], as well as dance movement therapy [88]. Moreover, therapies targeting sensorimotor and interoceptive processes show emerging efficacy for Black individuals with PTSD [89–91]. For example, trauma-focused yoga and aerobic exercise has been shown to be effective in remitting PTSD symptoms [92]. This is particularly relevant given that engagement in mind-body based interventions have been associated with functional changes in sensory processing networks. One study showed enhanced connectivity within the visual and SMN networks associated with better treatment outcomes in patients responsive to trauma-focused psychotherapy [93]. Moreover, given that functional connectivity within sensory somatomotor networks has been linked to treatment responsiveness in PTSD [93], the current findings also highlight the potential clinical relevance of targeting sensory and mind-body integration processes in trauma-related interventions such as interoceptive training, somatic therapies or neuromodulation for individuals exposed to RD.

We recognize some study limitations. Given the cross-sectional nature of the present study, we cannot make causal claims about the mechanisms underlying the associations between RD and lesser modularity and clustering of the SMN and its effects on PTSD symptomology. Longitudinal studies are needed to better characterize the mechanisms connecting experiences of RD to changes in the topological organization of the SMN and its effects on symptoms of re-experiencing. Another limitation is the usage of a specific parcellation scheme. We implemented a widely used network atlas but recognize that different parcellation schemes may elicit different modularity and clustering findings. Future studies employing multiple parcellation schemes are warranted to ensure replicability. Furthermore, we used a 20% threshold for significant connections to create our binarized adjacency matrix for network analysis. Indeed, prior studies have shown that adjusting the threshold can influence the topological organization of the networks rendering them more (or less) modular/clustered [94]. There is merit to utilizing multiple thresholds to examine potential effects on network topology in larger-scale studies. Lastly, only Black women were included in this study, precluding our ability to discern potential sex differences in findings. This sex homogeneity in our sample could also be considered a strength, as it may increase the sensitivity to detect effects that might be more difficult to observe in a mixed-sex cohort of similar size.

In summary, we observed that, even after accounting for other salient factors, greater self-reported RD was significantly associated with altered SMN topology. Greater RD exposure was characterized by greater de-differentiation of SMN architecture, in particular, diminished local clustering of the somatomotor network. This, in turn, is associated with greater severity of PTSD re-experiencing symptoms. These data suggest that altered SMN organization may represent a neural phenotype of racial discrimination and identity-related trauma, one that may be linked to wide-ranging functional consequences, from heightened sensory reliving of trauma to broader somatic disturbances such as chronic pain. Given that these trauma-related outcomes have long been documented in association with RD, findings illustrate the value of mind-body treatments that directly target sensory networks in racially marginalized groups.

## Supplementary Material

Supplementary Files

This is a filist of supplementary files associated with this preprint. Click to download.

• SupplementNMHElbasheir.docx

## Figures and Tables

**Table 1.** *Demographic and Clinical Characteristics* (N= 90)

Clinical Characteristic	Mean (SD) [Range]
Age (years)	38.49 (11.26) [18– 62]
Traumatic Event Exposure (TEI total)	4.58 (2.50) [0–11]
CTQ total	42.84 (18.14) [25–105]
PSS total	11.44 (11.16) [0–50]
EOD total	2.39 (2.21) [0–8]
Systemic Inequities Composite	0 (2.81) [−4.24–12.68]
Educational level*	% (N)
<12^th^ Grade	12.2 (11)
High school graduate or GED	32.2 (29)
Some college or technical school	40 (36)
College graduate	11.1 (10)
Graduate school	3.3 (3)
Monthly income***	% (N)
≤$249	11.1 (10)
$250-$499	11.1 (10)
$500-$999	32.2 (29)
$1000-$1999	25.6 (23)
≥ $2000	16.7 (15)

Abbreviations: EOD, Experiences of Discrimination Questionnaire; GED, General Educational Development certification; PSS, PTSD Symptom Scale; TEI, Traumatic Events Inventory.

* - missing 1 data point;

*** - missing 3 data point

**Table 2.** Partial Correlations Between Racial Discrimination and (A) Modularity and (B) Clustering Coefficient Network (including age, adult and childhood trauma exposure, systemic inequity and scanner type as covariates)

A								
	Total Discrimination	SMN Modularity	DMN Modularity	Dorsal Att Modularity	FPN Modularity	Limbic Modularity	Ventral Att Modularity	Visual Modularity
**Total Discrimination**	--	−.277	−.013	−.124	−.072	.156	−.058	−.057
**SMN Modularity**		--	.075	.011	−.111	−.140	.112	.091
**DMN Modularity**			--	.143	−.106	.156	.117	.219
**Dorsal Att Modularity**				--	−.001	.076	−.143	.120
**FPN Modularity**					--	−.080	.005	−.186
**Limbic Modularity**						--	−.012	.096
**Ventral Att Modularity**							--	−.011
**Visual Modularity**								--
(B)								
	Total Discrimination	SMN Clustering Coefficient	DMN Clustering Coefficient	Dorsal Att Clustering Coefficient	FPN Clustering Coefficient	Limbic Clustering Coefficient	Ventral Att Clustering Coefficient	Visual Clustering Coefficient
**Total Discrimination**	--	**−.318** *	−.098	−.060	−.080	−.216	.047	.053
**SMN Clustering Coefficient**		--	.075	.198	−.100	.079	.032	−.019
**DMN Clustering Coefficient**			--	.137	−.019	.166	.236	.010
**Dorsal Att Clustering Coefficient**				--	.161	**.337** *	.065	−.095
**FPN Clustering Coefficient**					--	.079	−.086	−.096
**Limbic Clustering Coefficient**						--	−.048	−.083
**Ventral Att Clustering Coefficient**							--	−.064
**Visual Clustering Coefficient**								--

Abbreviations: SMN, Somatomotor Network; DMN, Default Mode Network; Dorsal_att, Dorsal Attention Network; Ventral_att, Ventral Attention Network; FPN, Frontoparietal

* p < .007

**Table 3.** (A) Moderation Analysis with Racial Discrimination, SMN Clustering and Re-experiencing Symptoms (B) Conditional (+/- SD from the Mean) Effects of SMN Clustering Coefficient on Associations Between Racial Discrimination and PTSD Re-experiencing Symptoms (including age, adult and childhood trauma exposure, systemic inequity and scanner type as covariates)

(A)			
Predictor	β	p	95% Cl
Racial Discrimination (EOD total)	3.2	.02	[.38,6.12]
SMN Clustering Coefficient (CC)	8.84	.26	[−6.8,24.5]
RD (EOD total) x SMN CC	−5.4	.014	[−10.9, −.004]
Age	−.005	.85	[−.06,05]
Adult Trauma Exposure	.18	.17	[−.09,.46]
Childhood Trauma	.01	.68	[−.03,.05]
Systemic Inequities	.15	.17	[−.06,.36]
Scanner Type	.62	.35	[−.71, 1.9]
(B)			
SMN Clustering Coefficient Values	β	p	95% Cl
≤.48	.58	.002	[.22,.95]
.54	.30	.07	[−.02,.62]
≥.60	−.003	.98	[−.50, .50]

Abbreviations: SMN, Somatomotor Network; EOD, Experiences of Discrimination; CC, Clustering Coefficient; RD, Racial Discrimination
